# Ohio College of Dental Surgery, in Cincinnati

**Published:** 1845-06

**Authors:** Jesse W. Cook

**Affiliations:** Dean.


### ARTICLE VI.

We have just received the first annual announcement of the Ohio College of Dental Surgery, and as every effort to elevate the standard of professional qualifications must be a matter of interest to our readers generally, we publish it entire.—*Eds.*

*Ohio College of Dental Surgery, in Cincinnati.*—First Session, 1845–46.

A liberal charter has been granted by the legislature of this state, to establish a college in Cincinnati, with the above title.

The government of the institution is placed in the hands of the following board of trustees:

B. P. Aydelott, D. D., *President.*
Israel S. Dodge, M. D., *Secretary.*
Robert Buchanan,
Calvin Fletcher.
William Johnson,
J. P. Cornell, *of Cincinnati,*
G. S. B. Hempstead, M. D., *of Portsmouth,*
Samuel Martin, M. D., *of Xenia,*
James P. Hildreth, M. D., *of Marietta.*

### FACULTY.

JESSE W. COOK, M. D., D. D. S.,
*Professor of Dental Anatomy and Physiology.*
MELANCTHON ROGERS, M. D., D. D. S,
*Professor of Dental Pathology and Therapeutics.*
DR. JAMES TAYLOR, D. D. S.,
*Professor of Practical Dentistry and Pharmacy.*

Arrangements are in progress for the chemical chair, which will secure a thorough course in that department.

The first session of lectures in the institution will commence on the first Monday of November, and continue four months.

### TERMS OF ADMISSION.

The matriculating ticket will be $5 00. The ticket of each professor for the session will be $25 00. Dissecting ticket (optional) $10 00.

Diploma fee, $25 00. The fees for a full course will be $100 00, (exclusive of the diploma fee,) to be paid in advance.

### GRADUATION.

Candidates for graduation will be required to have attended two full courses of lectures, the last of which shall have been in this institution.

A full course of lectures in the Baltimore College of Dental Surgery, or a full course in a regular medical college, will be acknowledged as an equivalent to a course in this institution. The candidate must be twenty-one years of age, of good moral character, and have studied the profession two years with a reputable practitioner in dentistry.

A regular student of medicine, who has studied one year or more, and has taken a full course of lectures in a regular medical college, may be a candidate, after studying dentistry one year with a reputable practitioner, and taking one full course of lectures in this institution.

A reputable practitioner in dentistry who has been four years or more in practice, shall be entitled to an examination for a degree after attending one full course in this college.

Each candidate will be required to present and defend before the faculty, a written thesis on some subject relating to dental science, and be subject to a critical examination upon the theory and practice of dentistry.

### ANATOMY AND PHYSIOLOGY.

General and descriptive anatomy will be taught, in all its relations to dental surgery, by demonstrations on the subject, drawings, and preparations.

Physiological remarks will be made in connexion with anatomical demonstrations, so as to give to the latter additional weight and interest.

Anatomy, thus united with physiology, is here, as in medicine, the only groundwork of a correct dental education.

We are thus enabled to understand something of the translation of disease from one organ to another; to trace the effects of obstructed dentition to every sensible fibre; and to understand, also, the reason why artificial operations in mechanical dentistry so frequently fail.

That the dental student may have every opportunity of acquiring a thorough knowledge of anatomy, arrangements have been made with J. P. Judkins, M. D., as demonstrator in the dissecting-room, (which office he has held for the last six years in the Ohio Medical College.)

Independent of his regular anatomical demonstrations for this institution, Dr. Judkins, we understand, will deliver a private course of lectures on *descriptive and surgical anatomy*, to which the students of this college, who take the demonstrator's ticket, will be admitted without additional charge.

### DENTAL PATHOLOGY AND THERAPEUTICS.

It will be the province of this chair,

1st. To present a course of instruction upon the elementary principles of surgery, with such reference to general medicine as will enable the student to investigate the phenomena of diseases and appreciate their influences, directly and indirectly, upon the diseases of the mouth and teeth.

To attend profitably this part of the course, it is essential that the student shall have carefully read some of the standard elementary works upon general surgery and practical medicine; as most of the works on dentistry pre-suppose the student to have a knowledge of the general principles of medical science.

2d. To give a systematic course upon the diseases of the mouth and teeth, and the parts most intimately connected with them, embracing a critical investigation into their causes, as well as the nature and application of the modes of cure.

For this part of the course the student should have previously studied our best standard works on dental surgery.

### PRACTICAL DENTISTRY.

Every effort will be made to advance the student in this department of dental science. All the various operations will be performed before the class, and each student required to go through with all the manipulations of mechanical, as well as operative dentistry. Arrangements are being made which will give superior advantages for the acquisition of this part of the profession.

Practical knowledge, so important to the dentist, should, in all cases, be obtained before assuming the responsible duties of a practitioner. In dental offices, but limited opportunity for acquiring such knowledge is generally afforded. To meet this difficulty and present to the dental student an opportunity for a thorough medico-dental education, is the great object in the establishment of our institution. It is therefore intended that each student who pursues a regular course in the Ohio College of Dental Surgery, shall be enabled on leaving, to manufacture the teeth he requires for use, (particularly block teeth,) and also have some experience in the practical part of his profession.

### PHARMACY.

When we take into consideration the fact, that disease of the dental organs is generally induced by some chemical agent, the study of dental pharmacy assumes a magnitude but little appreciated in general practice. Such a course of lectures will be delivered on this subject as will enable the student to avoid the use of all improper and pernicious articles, in the various pharmaceutic preparations necessary to be used in practice; and, at the same time, direct the attention to such remedies as will remove, as far as possible, the proximate cause of disease.

The faculty of the Ohio College of Dental Surgery, in issuing this, their first announcement, do it with feelings of deep responsibility.

The establishment of a new institution for the diffusion of scientific knowledge, even under the most favorable auspices, is an undertaking of no small magnitude.

Every year makes it more and more apparent that some standard of dental attainment should be adopted—an intelligent public now require this. They feel that the continued impositions practised by the ignorant pretender need a remedy. The time has past, and we hope forever, when a little mechanical tact shall be considered sufficient to guide the dentist in his operations. There is no branch of operative surgery which demands more general knowledge. The student should continually bear in mind that his services will be required on a part of the general organization, the least derangement of which exerts an injurious effect on the whole of the system.

Daily observation verifies the fact that those who have most thoroughly studied dentistry, as a science, and have devoted most time and labor in preparing for its practice, never fail to be sustained by an enlightened public.

The monumental city in the east, and the queen city in the west, now claim the only dental colleges in the world—both are regularly chartered institutions, possessing full power to give instruction, confer degrees, &c. Their object is to secure the elevation of the dental profession and the relief of human suffering. It is with pleasure we can point to the east, and say, "The dawn of a better day" has opened upon us. We respectfully ask the profession of the west and a liberal public, if this light shall not be reflected?

The intelligent of the dental, as well as the medical profession, have been looking forward to the establishment of such an institution in the west, as one which the rapidly increasing population of this valley urgently demands. Every year adds more and more to the number engaged in practice, and each year, as the science keeps on in improvement,—its importance is raised in the estimation of the public; soon thousands instead of hundreds will be required in this department:—need we ask those who really feel an interest in its advancement, shall they be such as a confiding public may look to with safety for relief?

JESSE W. COOK, *Dean.*

